# A NEW MIXED SURGICAL TREATMENT FOR GRADES III AND IV HEMORRHOIDS: MODIFIED SELECTIVE HEMORRHOIDECTOMY COMBINED WITH COMPLETE ANAL EPITHELIAL RETENTION

**DOI:** 10.1590/0102-672020210002e1594

**Published:** 2021-10-18

**Authors:** Hua HUANG, Yunfei GU, Lijiang JI, Youran LI, Shanshan XU, Tianwei GUO, Minmin Xu

**Affiliations:** 1 Department of Anorectal, Changshu Hospital Affiliated to Nanjing University of Chinese Medicine, Changshu 215500, China; 2 Department of Anorectal, Affiliated Hospital of Nanjing University of Chinese Medicine, Nanjing 210000, China; 3 Nanjing University of Chinese Medicine, Nanjing 210000, China

**Keywords:** Severe mixed hemorrhoids, Anal pads, Anal canal epithelium, Complete anal canal retention, Hemorrhoid artery ligation, Milligan-Morgan, TST, Hemorroidas mistas graves, Almofadas anais, Epitélio do canal anal, Retenção completa do canal anal, Ligadura da artéria hemorroida, Milligan-Morgan, TST

## Abstract

**Background:**

Varicose veins appear above and below the dentate line in mixed hemorrhoids, which seriously affects anal function and quality of life.

**Aim:**

To propose an improvement in tissue-selecting therapy repair of anal pad combined with complete anal canal epithelial retention comparing with Milligan-Morgan surgery.

**Methods:**

A prospective randomized controlled study was designed enrolling 200 patients with grade III and IV hemorrhoids. They were divided into control and observation groups. The control received Milligan-Morgan surgery, and the observation the modified tissue-selecting therapy stapler combined with complete anal canal preservation surgery. All patients were followed for six months to evaluate the treatment differences.

**Results:**

In final, control group included 82 and observation 87. The average operation time of the control group was significantly lower than that of the observation, while the bleeding volume was significantly lower in control group. The control group VAS score was 3 (1, 4), and observation 4 (2, 5). There was no significant difference in the incidence of urinary retention, bleeding and wound margin edema after surgery at one month postoperatively. Digital incidence of anal stenosis in the observation group was significantly lower than in control; the same occurred with residual anal margins. The postoperative anal canal diameter was significantly larger than the control group. Wexner anal incontinence score showed that no anal incontinence occurred in both groups, and the control group scored was significantly higher than observation. In final six months follow-up, the observation group did not experience any relapse and four cases were found among controls. The treatment satisfaction of the observation group was better.

**Conclusions:**

In grades III and IV hemorrhoids, modified tissue-selecting therapy combined with complete anal canal preservation had better prognosis and treatment satisfaction than Milligan-Morgan procedure, and it is a new surgical method for patients with advanced mixed hemorrhoids.

## RESUMO

**Racional:**

Veias varicosas aparecem acima e abaixo da linha dentada nas hemorroidas mistas, afetando seriamente a função anal e a qualidade de vida.

**Objetivo:**

Propor melhoria na terapia de seleção de tecido de reparo do coxim anal combinado com retenção completa epitelial do canal anal em comparação com a operação de Milligan-Morgan.

**Métodos:**

Estudo prospectivo randomizado controlado foi desenhado envolvendo 200 pacientes com hemorroidas graus III e IV. Eles foram divididos em grupos de controle e observação. O controle recebeu operação de Milligan-Morgan, e o de observação procedimento de seleção de tecido modificado combinado com operação completa de preservação do canal anal. Todos os pacientes foram acompanhados por seis meses para avaliar as diferenças de tratamento.

**Resultados:**

No final, o grupo controle incluiu 82 e o de observação 87. O tempo médio de operação do grupo controle foi significativamente menor do que o de observação, enquanto o volume de sangramento foi significativamente menor no grupo controle. O escore VAS do grupo controle foi 3 (1, 4) e no de observação 4 (2, 5). Não houve diferença significativa na incidência de retenção urinária, sangramento e edema da margem da ferida no pós-operatório de um mês. A incidência de estenose anal digital no grupo observação foi significativamente menor do que no controle; o mesmo ocorreu com as margens anais residuais. O diâmetro do canal anal pós-operatório foi significativamente maior nele do que o grupo controle. A pontuação de incontinência anal de Wexner mostrou que nenhuma incontinência ocorreu em ambos os grupos, e a pontuação do grupo de controle foi significativamente maior do que no de observação. Nos últimos seis meses de acompanhamento, o grupo observação não teve nenhuma recaída e quatro casos foram encontrados entre os controles. A satisfação com o tratamento do grupo observação foi maior.

**Conclusões:**

Nas hemorroidas graus III e IV, o tratamento de seleção de tecido modificado combinado com a preservação completa do canal anal teve melhor prognóstico e satisfação do que com o procedimento de Milligan-Morgan, e é um novo método cirúrgico para pacientes com hemorroidas mistas avançadas.

## INTRODUCTION

Hemorrhoids are submucosal vascular tissues located in the anal canal. Symptoms include bright red bleeding from the anus and intestines, mucus discharge, perianal irritation or itching, pain around the anus, hemorrhoid pad prolapse or protruding masses, stains on underwear^12^. Global epidemiological studies have shown that hemorrhoids affect 4.40% of the world’s population; the global incidence is about 49.14%^17^ and is the most common anorectal disease in the world. In China, adults with anorectal diseases account for 51.14% of the total surveyed population, with the highest incidence rate of hemorrhoids (50.28%)^25^. A cross-sectional study^9^ pointed out that there is a widespread delayed treatment of hemorrhoids in China. In England, nearly hemorrhoids can be found in 40% of the screening colonoscopies performed^13^. In the U.S more than 2.2 million patients are seen in the clinic department every year^4^. Sandler’s study^18^ believed that although hemorrhoids are the cause of huge economic losses and personal suffering, it is surprisingly that they receive little research attention.

The pathological mechanism of internal hemorrhoids is the supporting structure of the anal cushion (anal canal vascular cushion), pathological changes and displacement of the vascular plexus and arteriovenous anastomosis^8^. The pathological mechanism of external hemorrhoids is the expansion of the subcutaneous vascular plexus in the distal dentate line, blood flow stasis, thrombosis or tissue hyperplasia^27^. According to the pathological characteristics of tissues, external hemorrhoids can be divided into connective tissue, thrombotic, varicose and inflammatory external hemorrhoids. Mixed hemorrhoids are internal and the external hemorrhoid vascular plexus of the corresponding site mutual fusion^22^. They are classified according to the degree of prolapse^14^ were grade III is prolapsed hemorrhoids that only require manual reduction and grade IV non-resettable ones. Hemorrhoids I and II are mainly mixed encouraging conservative treatment and for grades III and IV is require surgical treatment. 

At present, the mainstream traditional surgical methods for treatment hemorrhoids are open (Milligan-Morgan) and closed (Ferguson) hemorrhoidectomies ^7^ ^,^ ^23^. New surgeries and surgical instruments were designed and include LigaSure^TM^, Harmonic^®^ and Starion^TM,4.24^. Hemorrhoidal staples include stapler hemorrhoidal mucosal ring incision and staple surgery (PPH), selective superior hemorrhoidal mucosal nailing (TST), transanal stapler rectal resection (STARR)^29^ ^,^ ^30^.

With the dentate line as the boundary, the rectal column area about 1.5 cm above the dentate line is the anal pad. In the past, the treatment of anal pad in traditional surgery was “destructive”, even if the damage was large or small. Although the surgery achieved good results, however it has a greater impact on the protection of anal canal function and the quality of life of patients after surgery^15^ ^,^ ^28^. The proposed anastomosis surgery theoretically will not invade the anal cushion, but it has a higher recurrence rate and more post-complications^10^ ^,^ ^21^. Therefore, how to protect the anal cushion to the greatest extent while ensuring the efficacy, it has become the focus of internal hemorrhoid surgery. The anal canal epithelium below the dentate line is composed of squamous epithelium. No regeneration function after skin defects^5^. Defects of the anal canal epithelium can cause scar hyperplasia and cause anal stenosis. Anal canal epithelium is innervated by pain-sensitive sacral nerves. When the anus is stimulated by the outside, it can cause muscle spasm and produce severe pain. In addition, excessive anal canal epithelial damage can also cause closed dysfunction due to anal exudate and decreased anal sensory sensitivity, secretions cannot be controlled, as anal dampness and other complications^1^. Therefore, how to effectively treat is to keep, as much as possible, anal canal epithelium that has important clinical significance.

Based on the current status of surgical treatment of grade III and IV hemorrhoids, this study aims to propose an improvement in TST repair of anal pad combined with complete anal canal epithelial retention (CACP), comparing it with Milligan-Morgan procedure. 

## METHODS

The study project was approved by the Clinical Ethics Committee of the Changshu Hospital Affiliated to Nanjing University of Chinese Medicine. All patients were informed of its content and signed informed consent.

### Patients

A prospective randomized controlled study was designed. Two hundred patients with hemorrhoids who underwent surgical treatment from June 2017 to June 2019 were selected. Inclusion criteria were: 1) clinical diagnosis of mixed hemorrhoids and Banov classification of internal hemorrhoids grades III and IV^14^; 2) symptomatic external hemorrhoids; 3) age between 18 and 70 years; 4) first mixed hemorrhoid surgery; 5) no anal morphological and functional abnormalities. Exclusion criteria were: 1) have had mixed hemorrhoids surgery, or other perianal disease surgery; 2) pregnancy, breastfeeding and women during menstrual and menstrual periods; 3) functional impairment of important solid organs, such as liver and kidney; 4) rectal cancer, rectal polyps, tuberculosis, Crohn’s disease and other rectal and anal diseases; 5) severe diseases of blood circulation, blood system; 6) acute inflammatory or thrombotic external hemorrhoids. 

### Preoperative preparation

Enrolled patients were submitted to complete examination to confirm their conditions to the procedures. If contraindication existed, they were submitted to other diagnostic examinations to reinforce their inclusions. They were randomly divided into a control group (Milligan-Morgan surgery) and an observation group (modified TST combined with CACP surgery) according to the random number table method. After determining the time of the operation an enema 8 h in advance realized and the circumference of the anal canal was measured. All patients were placed in the lateral position and underwent to epidural anesthesia.

### Milligan-Morgan surgery

According to the shape of the hemorrhoid, the segment and quantity of the anal canal cutaneous bridge and mucosal bridge were designed. A V-shaped incision from the skin of the anal margin were made, and gradually separated it to the dentate line along the surface of the internal sphincter. The internal hemorrhoids, external hemorrhoids, and perianal skin were clamped up so that the three were in line. Thin and long radial incision from outside to inside to the dentate line was made. According to the size of the hemorrhoid core, appropriate vascular forceps were used to clamp the bottom of the internal hemorrhoid base. “0” thread was used to sew, and the hemorrhoid tissue was cut off above and beyond the knot. Part of the varicose veins of the external hemorrhoids, as well as the connective external hemorrhoids tissue, were directly removed.

### Modified TST combined with CACP surgery

TST surgical instruments were open-loop minimally invasive hemorrhoidal staplers. Single-, double-, or triple-opening anoscopes were selected based on the number, size, location, and distribution of internal hemorrhoids to fully expand the anus. Anal mirrors with lubricated paraffin oil were placed in the anus.

The inner tube removed and the anoscope adjust to make the mucosa of the hemorrhoid which needed to be closed was fully exposed in the window. At 2 cm above the dentate line, a 7-gauge silk thread was used for a mucosal and submucosal segmented purse suture. After being the stapler fully opened, the anvil head was placed in the anus above the purse suture site and passed through the purse suture. In order to get the mucosa into the stapler, the purse sutures were knotted and pulled. The anoscope were removed after the hemostasis were fully stopped. For the internal hemorrhoids not touched by the stapler, 1-2 ml of lauromacrogol injection (Shanxi Tianyu Pharmaceutical Co., Ltd., national drug approval No. h20080445, specification: 10 ml: 0.1 g/piece) was extracted with 5 ml syringe and injected into hemorrhoid mucosa and upper hemorrhoid mucosa. 

The two ends of the external hemorrhoid were clamped with vascular forceps, and a curved incision was made on the skin line of the anal canal with a scalpel. Stripped from the incision if severe varicose veins were found. The skin edge was trimmed to a flat, and mattress suture was performed with 3-0 absorbable thread. The incision was sutured with 4-0 absorbable thread at both ends of the incision. No bleeding was detected at the end of the operation, and pressure bandaging was performed; the resected tissue was sent to the pathology department for pathological examination.

### Postoperative management

The patient ate a normal diet after surgery and controlled defecation within 24 h; intravenous drip antibiotics to prevent infection and tranexamic acid to stop bleeding were used. Compound carrageenan suppositories were used every night to protect the wound in the anus, the wounds of external hemorrhoids were treated with routine auxiliary materials. After complete healing, the circumference of the anal canal was measured one month after.

### Follow-up and data collection

All patients had established independent case files to record all data. Was set up an electronic summary table to register the patient’s treatment information and observation index data. When the patient was hospitalized, was conducted a full communication study, and emphasized patient´s necessity on follow-up for six months after the operation. The medical records of all patients were written in detail. Was checked the correctness of the contact information again after the patient was discharged to reduce postoperative follow-up.

### Observing indexes & evaluation standard

#### Intraoperative

Surgery duration: Time from the completion of the operation of the surgical towel to the end of the operation, in minutes (min).

Intraoperative bleeding: Blood volume of each small square gauze soaked was 5 ml, and intraoperative bleeding volume was measured in milliliters (ml).

### Within one week after operation

1) Urinary retention: Twelve-hour postoperative period as the observation time to evaluate the patient’s active urination and corresponding treatment measures. Patient´s evaluation of the urinary retention, according to the difficulty of urination, was: who could urinate normally after surgery had grade I (0 points); who could urinate on their own but with difficulties had grade II (1 point); who needed assistance in urination had grade III (2 points); and the ones who had the need to maintain the catheter were evaluated as grade IV (3 points).
2) Postoperative bleeding: The first defecation after 48 h from surgery was unified recording time. Bleeding observation of the wounds in the two groups was recorded and treatment established according to the bleeding. Graduation was considered in relation to the bleeding amount in: no bleeding after surgery, grade I (0 points); with a small amount of bleeding (blood stains were only found in the toilet paper), grade II (1 point); blood dripping (bleed volume ≤10 ml), grade III (2 points); obvious blood dripping or even spurting (bleeding volume >10 ml), grade IV (3 points).
3) Incision pain: During the hospitalization, the patients were observed and recorded on the 3^rd^ day after surgery, and the degree of pain was recorded with visual analog scale (VAS), being no pain (VAS=0 points) and greater pain (VAS=10 points).
4) Wound margin edema: On the 5^th^ day after surgery, patients without anal margin edema were evaluated as grade I (0 points); with mild anal margin edema occupying 1/4 circle of perianal stamen as grade II (1 point); with anal marginal edema occupying more than 1/4 of the perianal circle and less than 1/2 circle as grade III (2 points); with anal marginal edema accounting for >1/2 of the perianal circle as grade IV (3 points).

#### One month after operation

1) Anal stenosis: The compliance and natural elasticity of the anal opening were lost, with fibrous shape abnormally tight and the index finger could not pass through the anus smoothly during digital examination.
2) Residual skin tags on the anal margin: Patients with smooth and flat anal areas and no skin tags were evaluated as grade I (0 points); with less than three local areas slightly convex and asymptomatic as grade II (1 point); three or more areas prominently raised and asymptomatic as grade III (2 points); with more than three locally raised bumps and asymptomatic as grade IV (3 points).
3) Anal canal circumference: Varicose external hemorrhoids. The diameter of the hemorrhoid core and the width of the incision were measured and recorded. The core diameter was selected from the anal margin line to the end of the hemorrhoidal core. The width of the incision was between the anal skin and the incision. Maximum distance in each patient was measured once before and after surgery. Postoperative measurements were performed after the patient’s internal hemorrhoids and hemorrhoids have all fallen off and the incision has completely healed. Generally, measurements were taken about one month after surgery.
4) Anal incontinence^16^: Wexner anal incontinence score was used to evaluate anal incontinence.

#### Six months after operation

1) Recurrence rate: Asymptomatic for at least two months after surgery, and then recurrence of symptoms was considered recurrence. The number of relapses within six months after surgery was counted, the cause of recurrence determined, and the corresponding treatment if done (if recurrence was diagnosed by a specialist, telephone follow-up combined with outpatient review was required).
2) Patient satisfaction: After six months, the patient evaluated the overall efficacy of the treatment using a percentage system divided into 0-20 points, 21-40 points, 41-60, 61-80 points, and 81-100 points.

### Statistical analysis

SPSS 24.0 (SPSS Inc. Chicago, IL, USA) was used for statistical analysis of the data. The count data was represented by examples (percent, n%), the theoretical number T ≥5 and the total sample size n ≥40 using Pearson χ^*2*^ test; theoretical number T <5 but T ≥1, and n ≥40, using the continuous correction of χ^*2*^ test; theoretical number T <1 or n <40, use Fisher’s exact test. The measurement data obeying normal distribution were expressed as mean±standard deviation (± s) and indicated that the comparison between groups was performed with an independent sample t test, and the comparison within a group performed with a paired sample t test. Data that do not obeyed the normal distribution were expressed as P_50_ (P_25_, P_75_). Mann-Whitney U test was used for comparison. Mann-Whitney U test was also used for the difference in the distribution of grade data. Both levels of α=0.05 were used for the test level, and p<0.05 was evaluated as a statistically significant difference.

## RESULTS

### Baseline data of patients

According to the inclusion and exclusion criteria, the first 200 patients were included in the study. The control group and the observation group recruited 100 patients each as expected. During the six-month follow-up period, the control group lost 18 patients and the observation group 13. Therefore, a total of 169 patients were finally included in the study, 82 in the control group and 87 in the observation group. There was no significant difference in baseline data between the two groups (p>0.05) (Table 1).

**TABLE 1:** Baseline data for two groups of patients

Variables	Control group	Observation group	χ2/t value	p
Cases (n)	82	87		
Age (years)	48.1±13.7	46.8±17.5	0.539	0.590
Gender (n)				
male	29	33	0.120	0.729
female	53	54		
BMI (kg/m2)	23.8±2.9	23.3±3.2	1.065	0.288
Duration of disease (years)	13.5±9.3	12.6±10.4	0.594	0.554
Spicy eating habits (n)				
positive	14	17	0.172	0.679
negative	68	70		
Drinking habits (n)				
positive	17	19	0.031	0.861
negative	65	68		
Sedentary habit (n)				
positive	35	40	0.186	0.667
negative	47	47		
Family history (n)				
positive	42	45	0.004	0.948
negative	40	42		
Staging of internal hemorrhoids (n)				
grade III	55	51	0.120	0.793
grade IV	27	36		

BMI=Body Mass Index

### Intraoperative observation

**FIGURE 1:**
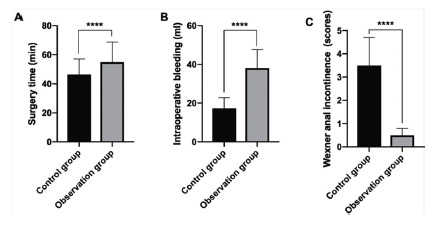
Comparisons between two groups of patients: A) operation time; B) intraoperative blood loss; C) anal incontinence scores.

The minimum operation time in the control group was 35 min and the longest 70 min. The minimum operation time in the observation group was 45 min and the longest was 80 min. The average operation time in the control group was significantly lower than that in the observation group (46.4±10.7 vs. 54.9±13.8, p<0.0001, Figure 1A). The intraoperative blood loss in the control group was at least 30 ml and at most 58 ml. The intraoperative blood loss in the observation group was at least 10 ml and at most 32 ml. The intraoperative blood loss was significantly lower than that in the control group (17.3±5.5 vs. 38.1±9.6, p<0.0001, Figure 1B).

### Observation within one week postoperatively

All patients had postoperative VAS scores ranging from 1-6, and the two groups of data did not conform to a normal distribution. The control group had a VAS score of 3 (1, 4) and the observation VAS of 4 (2, 5). Mann-Whitney U test showed no significant difference in VAS scores between the two groups of patients (p>0.05).

As shown in Tables 2, 3 and 4, 55 patients in the control group did not experience urinary retention, 46 no postoperative bleeding, and 58 didn´t have anal marginal edema; 58 patients in the observation group did not experience urinary retention, 55 had no postoperative bleeding, and 60 cases had no anal marginal edema. According to the evaluation criteria, patients’ urinary retention, postoperative hemorrhage, and wound marginal edema were classified according to grades I-IV. Mann-Whitney U test showed that there was no significant difference in the occurrence of urinary retention, bleeding, and wound edge edema in two groups of patients (p>0.05).

**TABLE 2:** Two groups postoperative urinary retention

Grade	Control group	Observation group	U value	p value
I	55	58	0.855	0.327
II	22	18		
III	4	5		
IV	1	2		

**TABLE 3:** Two groups postoperative bleeding

Grade	Control group	Observation group	U value	p value
I	46	55	0.593	0.280
II	26	20		
III	10	12		
IV	0	0		

**TABLE 4:** Two groups postoperative wound margin edema

Grade	Control group	Observation group	U value	p value
I	58	60	0.541	0.407
II	17	18		
III	7	9		
IV	0	0		

### Observation in one month postoperatively

The patient underwent digital anal examination during the follow-up of one month after the operation. No anal stenosis occurred in the observation group, and four cases occurred in the control. The incidence of anal stenosis was significantly lower in the observation group than in the control (p<0.05). In the observation group, 71 patients had no residual anal marginal skin tags, and 55 in controls had no residual anal marginal skin tags. According to the evaluation criteria, the patient’s residual anal marginal skin tags were classified according to the grade I-IV. Whitney U test showed that the residual anal margins in the observation group were more advantageous than those in the control group (p<0.05, Table 5). As shown in Table 6, the preoperative anal canal diameter of the observation group was significantly lower than that of the control group. There was no significant difference (8.34±0.35 vs. 8.38±0.39, p=0.485). The postoperative anal canal diameter in the observation group was significantly larger than that in the controls (8.01±0.29 vs. 7.55±0.32, p<0.0001). Wexner anal incontinence score showed that there was no complete anal incontinence in the two groups, and the scores in the control group were significantly higher than those in the observation one (3.5±2.1 vs. 0.5±0.3, p<0.0001, Figure 1C).

**TABLE 5:** Two groups postoperative residual skin tags

Grade	Control group	Observation group	U value	p value
I	55	71	3.216	0.023
II	20	12		
III	7	2		
IV	0	0		

**TABLE 6:** Two groups changes on anal canal diameter before and after surgery

Variables	Control group	Observation group	t value	p value
Pre-operation	8.38±0.39	8.34±0.35	0.700	0.485
Post-operation	7.55±0.32	8.01±0.29	9.801	<0.0001
t value	15.074	6.689	/	/
p value	<0.0001	<0.0001	/	/

**TABLE 7:** Two groups satisfaction survey

Score	Control group	Observation group	U value	p value
0-20	0	0	2.367	0.011
21-40	0	0		
41-60	9	1		
61-80	23	20		
81-100	50	66		

### Observation in six months postoperatively

During the six months of follow-up, there were no cases of recurrence in the observation group and four in the control group. Fisher’s exact test showed no significant difference in the recurrence rate between the two groups (p=0.053). However, the treatment in the observation group was satisfactory and more advantageous than the control group (p<0.05, Table 7).

## DISCUSSION

Milligan-Morgan surgery is considered to be the “gold standard” for hemorrhoid surgery, and it has been used as a control method in many studies^2^ ^,^ ^11^. This study compared our new method with Milligan-Morgan surgery, and RCT study was designed. The research results show that Milligan-Morgan surgery has the advantage of shorter operation time. There is no significant difference in VAS scores between the two groups of patients. In addition, the two groups have no significant difference in the incidence of postoperative urinary retention bleeding and wound margin edema. However, the modified TST and CACP procedure showed more advantages in patient prognosis. Patients treated with the combination procedure had lower intraoperative blood loss. One month after surgery at follow-up, the combined operation had a lower incidence of anal stenosis, fewer residual anal marginal skin tags, larger anal canal circumference, and better anal function. The patient satisfaction on the combined operation was significantly better than Milligan-Morgan. This may be related to the improvement of the patient’s prognosis. The superiority of the combined procedure is related to the advantages of the modified TST and the first proposed CACP procedure.

The observation group used modified TST to remove internal hemorrhoids. TST surgery is a new type of surgical technique developed on the basis of PPH surgery^30^. In TST the mucosa is not resected annularly, but only through a specially opened anus Mirrors (single, double, and triple openings) selectively remove the prominent mucosa of the hemorrhoids while retaining the normal mucosa between the resected mucosa. Compared with PPH surgery, it reduces the overreaction of staples and tissues. On the other hand, the retained mucosal bridge forms an elastic annular rubber band on the anastomosis surface, which enables better rectal compliance during defecation^19^. However, the recurrence rate and cause of recurrence are similar to those of PPH^3^. The research of TST technique is still in the exploratory stage. On the one hand, there are few high-quality clinical RCT studies on the other hand and no uniform surgical operation standard. The improved TST technique is carried out by selecting 2 cm of dental line for purse suture. During the partial resection and suspension of internal hemorrhoid lesions, the normal mucosa between the resected mucosa is well preserved, and the untreated internal hemorrhoids are injected with local sclerosing agent^20^ ^,^ ^6^. Modified TST surgery is different from previous treatment of internal hemorrhoids, it repairs the anal pad while protecting the anal function to the greatest extent.

For the treatment of external hemorrhoids in observation group, the CACP technique, which we designed through many years of clinical experience, is used for the first time. The surgery is performed by making an arc incision on the anal canal skin margin, conformally removing the external hemorrhoids, and making varicose veins. Undercover peeling and suture the incision to completely protect the anal canal epithelium. Through the design of many details of the operation, the purpose of fast wound healing, fewer postoperative complications, low recurrence rate, and high patient satisfaction is achieved. The protection of function is getting more and more attention and improving the cure rate under the premise of protecting the anal function is the focus of future anorectal surgery research. Takano Masahiro^26^ is a widely recognized anal canal epithelial retention surgery in Japan proposed in 1989 based on the dorsal anal cushion doctrine theory, which cuts the external and internal hemorrhoids slightly wider, and cuts the anal canal slightly narrower, and the entire incision is dumbbell-shaped, keeping as much anal canal epithelium as possible. There are few clinical reports about the anal canal epithelium protection in the mainstream surgical procedures at home and abroad, or although there are reports, no complete preservation of the anal canal epithelium or the elaboration of the operation is not clear. The CACP technique is a method to completely protect the anal epithelium, and the operation procedure has been provided in this thesis.

This study summarizes the key points of CACP: 1) the inner edge of arc resection of external hemorrhoids is the anal skin line, and the outer edge is the outer margin of external hemorrhoids; however, the incision width should not be too wide, which will lead to the risk of excessive tension; 2) 3-0 Vicryl suture is used as the main framework of incision, and the suture should not be too dense; 4-0 fast Vicryl suture is used at both ends and suture gap, which can not only strengthen the firmness of the incision, but also avoid postoperative scar caused by large incision, reduce postoperative incision edema and discomfort, and promote wound healing; 3) when suturing the incision, the skin edge should be trimmed to make the skin in a good position; mattress suture should be performed; and, the knot should be located at the outside edge of the skin to avoid the contact between the suture knot and the anal canal skin; 4) varicose vein mass should be exfoliated before stitching. 

There are still limitations in this study. It is a single-center research, and the number of patients ultimately included is small. It only conducted short-term follow-up (six months), and longer follow-up after this operation was not done. The control group was Milligan-Morgan procedure and, as there are other techniques, further researches must be done to compare our proposal with existent different surgeries, in future.

## CONCLUSIONS

The modified TST combined with CACP operation not only effectively protects and repairs the anal cushion, but also completely preserves the patient’s anal canal epithelium. Therefore, this first proposed technical combination can more effectively protect the function of the anus. In grades III and IV hemorrhoids it had better performance in prognosis and treatment satisfaction than Milligan-Morgan procedure. It is a new surgical method for patients with advanced mixed hemorrhoids.
